# Development of mucosal‐associated invariant T cells

**DOI:** 10.1111/imcb.12039

**Published:** 2018-04-24

**Authors:** Hui‐Fern Koay, Dale I Godfrey, Daniel G Pellicci

**Affiliations:** ^1^ Department of Microbiology and Immunology Peter Doherty Institute for Infection and Immunity University of Melbourne Melbourne VIC 3000 Australia

**Keywords:** Development of MAIT cells, mucosal‐associated invariant T cells, T‐cell receptor

## Abstract

Mucosal‐associated invariant T (MAIT) cells develop in the thymus and migrate into the periphery to become the largest antigen‐specific αβ T‐cell population in the human immune system. However, the frequency of MAIT cells varies widely between human individuals, and the basis for this is unclear. While MAIT cells are highly conserved through evolution and are phenotypically similar between humans and mice, they represent a much smaller proportion of total T cells in mice. In this review, we discuss how MAIT cells transition through a three‐stage development pathway in both mouse and human thymus, and continue to mature and expand after they leave the thymus. Moreover, we will explore and speculate on how specific factors regulate different stages of this process.

## Introduction

Mucosal‐associated invariant T (MAIT) cells are a highly conserved population of T lymphocytes that exhibit innate‐like functions similar to those described for natural killer T (NKT) cells.^1^, ^2^, ^3^, ^4^ MAIT cells are defined by their invariant T‐cell receptor (TCR)‐α chain, and their restriction to the nonpolymorphic major histocompatibility complex class I‐like molecule, MHC‐related protein 1 (MR1).^2^ In mice, MAIT cells express a TCR comprising of Vα19 joined to Jα33, which pairs with Vβ6 or Vβ8.^5^, ^6^, ^7^ In humans, MAIT cells predominantly express Vα7.2 joined to Jα33, Jα20 or Jα12, and these pair with Vβ2 or Vβ13.^3^, ^5^, ^7^ Smaller subsets of MR1‐restricted Vα7.2^−^ T cells have been identified in humans,^8^, ^9^, ^10^ although this review will focus on the major population of Vα7.2^+^ MAIT cells, herein simply described as MAIT cells.

In this issue, Lantz and Legoux^11^ provide a historical perspective of the MAIT cell field, including the initial identification and the description of MAIT cells as “mucosal‐associated” due to their presence within the gut lamina propria.^2^ It is now appreciated that MAIT cells can be found in all sites where conventional T cells are present,^6^, ^12^, ^13^ although functionally distinct subsets of MAIT cell exist at mucosal sites, suggesting an important role for these cells in barrier tissue immunity.^12^, ^14^ MAIT cells are abundant in humans, making up to 10% of T cells in peripheral blood and up to 50% in the liver,^14^, ^15^ yet their frequency can significantly vary between individuals.^13^, ^15^, ^16^, ^17^ In mice, the frequency of MAIT cells is much lower, and it is currently unclear what factors regulate MAIT cell numbers and cause this disparity between species.^6^, ^7^, ^16^ Our understanding of human MAIT cells has progressed predominantly through the study of CD3^+^ Vα7.2^+^ CD161^hi^ T cells, which incorporates most MAIT cells in peripheral blood and organs.^13^, ^18^, ^19^ Conversely, because MAIT cell numbers in laboratory mice are very low, and monoclonal antibodies against the invariant mouse MAIT TCRα chain do not exist, Vα19Jα33 transgenic mice were established to investigate the biology of mouse MAIT cells.^16^, ^20^ The discovery of riboflavin derivatives as antigens (Ags) for MAIT cells^21^, ^22^ (described in this issue by Kjer‐Nielsen, Corbett and colleagues^23^), coupled with the production of MR1 tetramers that incorporate these Ags,^5^ has fueled new studies into the development and function of MAIT cells. These tetramer reagents now provide a basis for distinguishing MAIT cells from other T‐cell populations that share similar phenotypic markers.^8^, ^9^, ^24^, ^25^

MR1 is expressed at low levels in a wide range of tissues^26^, ^27^ and shows strong evolutionary conservation between species.^28^ This, together with the broad microbial reactivity of MAIT cells suggests that they play a key role in host immunity.^29^, ^30^, ^31^, ^32^ The role that MAIT cells play in microbial immunity is extensively reviewed in this issue by Ussher, Willberg and Klenerman (MAIT cells and Viruses)^33^ and Meermeier, Harriff, Karamooz and Lewinsohn (MAIT cells and Microbial Immunity).^34^ Given the wide variation in MAIT cell frequency between individuals^13^, ^16^, ^19^, ^35^ and evidence that reduced MAIT cell numbers are associated with aberrant immune responses in humans and mice,^29^, ^30^, ^31^, ^36^, ^37^, ^38^, ^39^ it is important to understand how MAIT cells develop and determine what factors regulate this process. In this review, we describe how MAIT cells undergo a three‐stage development pathway within the thymus and continue to mature after they enter the periphery, in both mice and humans. In addition, we examine similarities and differences with the development of NKT cells, which may compete with MAIT cells for a similar environmental niche.

## Thymic Selection

Studies in mice revealed that MAIT cells arise intrathymically following interaction of the MAIT TCR with MR1.^2^, ^7^, ^16^, ^40^ Furthermore, analysis of Vα19‐Jα33 Cα^−/−^ and Vβ6 transgenic mice revealed that MAIT cells are selected by MR1‐expressing DP cortical thymocytes, compared to thymic epithelial cells that select MHC/peptide reactive T cells.^40^ It is currently unclear whether specific Ags must be presented in association with MR1 for intrathymic selection, and if so, the nature of the selecting Ags. Selection may require agonist ligands like the MAIT activating riboflavin derivative Ags such as 5‐(2‐oxopropylideneamino)‐6‐d‐ribitylaminouracil (5‐OP‐RU) and 5‐(2‐oxoethylideneamino)‐6‐d‐ribitylaminouracil (5‐OE‐RU).^22^ Alternatively, MAIT cells might be selected by a weak or nonagonist Ag, such as 6‐formylpterin (6‐FP), a vitamin B9 derivative that binds to MR1 but does not activate the majority of MAIT cells.^21^, ^41^ Although these vitamin‐derivative Ags have microbial or dietary origins, it is also possible that MAIT cells are selected in the context of an endogenous self‐Ag produced in the thymus. Early evidence supported a role for microbial derived Ags for the selection of MAIT cells as these cells could not be detected in germ‐free (GF) mice using RT‐PCR.^2^ We recently re‐examined GF mice using MR1 tetramers and detected the presence of normal numbers of immature MAIT cells in the thymus, but these cells were unable to fully mature and expand in the periphery.^24^ These data suggest that microbial colonization is not required for the initial selection of MAIT cells, but is essential for their subsequent expansion.^16^, ^24^ Another important question surrounding the selection of MAIT cells is whether their TCR repertoire is shaped by negative selection. Like MAIT cells, NKT cells are selected by DP cortical thymocytes,^42^ and *in vivo* injection of the NKT cell agonist Ag α‐galactosylceramide or the addition of this lipid Ag to fetal thymic organ cultures ablated the development of mouse NKT cells, suggesting these cells had undergone negative selection.^43^, ^44^ It will be important to establish if similar selection criteria also exist for MAIT cells. For instance, would the overexpression of MR1 or the presence of a high affinity Ag lead to the deletion of MAIT cells? Accordingly, further studies are required to examine the types of Ags (if any) that govern the intrathymic selection of MAIT cells.

## A Three‐Stage Pathway for MAIT Cell Development in Mice and Humans

Analysis of MAIT cells from the periphery of WT mice using MR1‐5‐OP‐RU tetramers revealed that they expressed high levels of CD44 and had a memory phenotype, whereas most MAIT cells from Vα19‐Jα33 Cα^−/−^ transgenic mice lacked CD44 expression and were described as naïve.^6^, ^16^ Moreover, and in contrast to previous findings,^16^ MAIT cells from WT mice expressed the transcription factor, promyelocytic leukemia zinc finger (PLZF).^6^, ^45^ PLZF was previously reported to be required for the development of other innate‐like T cells such as NKT cells,^46^, ^47^ innate lymphoid cells^48^, ^49^ and some γδ T cells.^50^, ^51^ These data highlight important differences in the phenotype of MAIT cells from WT and Vα19 transgenic mice and suggest that the overexpression of the mouse MAIT TCR α‐chain likely alters the development of MAIT cells.

**Figure 1:**
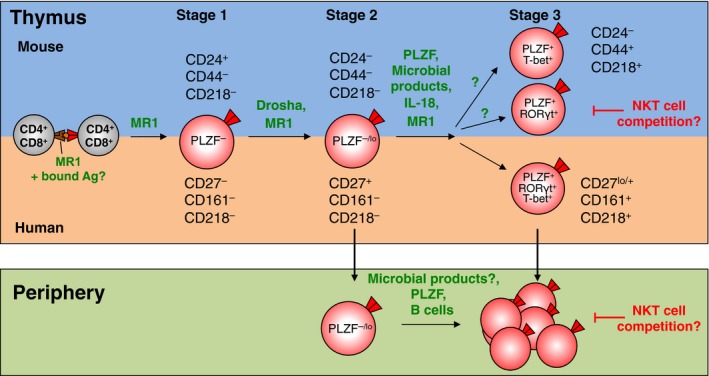
A three‐stage pathway for MAIT cell development in mice and humans. Competition between MAIT cells and NKT cells for a shared niche in mice.

Our studies of mouse thymus revealed three populations of MAIT cells based on their expression of CD24 and CD44, including CD24^+^CD44^−^, CD24^−^CD44^−^ and CD24^−^CD44^+^ MAIT cells.^24^ Through a combination of phenotypic analysis, ontogeny experiments and *in vitro* development studies, we determined that the CD24^+^CD44^−^ population were least mature, defined as stage 1 MAIT cells. These give rise to CD24^−^CD44^−^ stage 2 cells and ultimately these differentiate into CD24^−^CD44^+^ stage 3 cells, which more closely resemble MAIT cells in the periphery (Figure 1). Importantly, MR1 expression appears to be required at each stage of development, as progression from stage 1 to stage 3 *in vitro*, is severely impaired by the addition of an anti‐MR1 blocking antibody.^24^ These distinct MAIT cell precursor populations were further characterized for expression of differentiation markers, cytokine receptors and transcription factors (Table 1). For example, PLZF is not expressed in stage 1 MAIT cells, while mature populations of stage 3 MAIT cells express PLZF.^24^ This contrasts with the development of NKT cells, as early NKT cell progenitors express high levels of PLZF, and expression levels decline to moderate levels as NKT cells become more mature, with the notable exception of NKT2 cells that express high levels of PLZF (discussed below).^46^ Analysis of PLZF‐null mice revealed that the development of stage 1 and 2 MAIT cells was unperturbed, while mature stage 3 cells failed to develop within the thymus and periphery. Moreover, residual MAIT cells from PLZF‐null mice were unable to produce cytokines and were hence functionally incompetent.^24^ Therefore, PLZF is a critical factor governing the transition from stage 2 to stage 3 MAIT cells, as well as the acquisition of MAIT cell functional potential.

**Table 1:** Phenotypic characteristics of stage 1, stage 2 and stage 3 thymic MAIT cells in mice and humans

	Stage 1	Stage 2	Stage 3
CD4 and CD8 coreceptor	CD4^+^, CD8^+^ or CD4^+^CD8^+^	h: CD4^+^, CD8^+^ or CD4^+^CD8^+^	CD4^+^, CD8^+^ or
		m: CD4^+^, CD8^+^ or CD4^−^CD8^−^	CD4^−^CD8^−^
CD8αβ	CD8αβ^+^	CD8αβ^+^	CD8αβ^+^
CD24 (m)	Positive	Negative	Negative
CD27 (h)	Negative	Positive	Low to positive
CD44 (m)	Negative	Negative	Positive
CD62L (m)	Intermediate	n.d.	Negative
CD69 (m)	Positive	n.d.	Negative
CD103 (m)	Negative	n.d.	Low to positive
CD161 (h)	Negative	Negative	Positive (h)
NK1.1 (m)			Low to positive (m)
CD278/ICOS (m)	Negative	n.d.	Positive
Cytokine receptors			
CD122/IL‐2Rβ (m)	Negative	n.d.	Intermediate
CD127/IL‐7R (m)	Negative	n.d.	Positive
CD218/IL‐18R	Negative	n.d.	Positive
Transcription factors			
PLZF	Negative	Low	Positive
T‐bet and RORγt	Low	Low	T‐bet^+^ or RORγt^+^ (m) T‐bet^+^ RORγt^+^ (h)

n.d. = not determined; m = mouse; h = human.

Most studies on MAIT cell development in humans have utilized surrogate markers, such as Vα7.2 or co‐expression of Vα7.2 and CD161, to identify MAIT cells. Using this strategy, MAIT‐like cells could be detected within human thymus and were shown to express PLZF.^16^, ^52^, ^53^ Moreover, Leeansyah and colleagues examined a panel of human fetal tissues including the thymus and cord blood, and described a maturation pathway of microbial reactive Vα7.2^+^ CD161^+^ cells.^52^ Our laboratory examined MAIT cells from human thymus using MR1 tetramers and similar to our mouse studies, we identified three populations of MAIT precursors based on the differential expression of cell surface markers.^24^ Ontogeny and functional studies showed that stage 1 human MAIT cells could be defined as CD27^−^CD161^−^, followed by stage 2 CD27^+^CD161^−^ and then stage 3 CD27^lo/+^CD161^+^ (Figure 1 and Table 1). Moreover, the expression of CD161 on MAIT cells from human thymus also coincided with the expression of IL‐18R, the latter expressed by stage 3 thymic MAIT cells from both humans and mice (Table 1).^24^ Notably, the vast majority of human thymic MAIT cells lack expression of CD161 and these cells would have been excluded from previous studies that relied on CD161 as a surrogate marker of MAIT cells.^16^, ^24^, ^52^ In addition, we also found many MAIT‐like Vα7.2^+^ CD161^+^ T cells in human thymus that do not bind MR1‐5‐OP‐RU tetramer, a result that was recently observed by another group with human cord blood and neonatal blood samples.^25^ The majority of peripheral blood MAIT cells have upregulated CD161 and IL‐18R, although residual CD161^−^ IL‐18R^−^ MAIT cells could still be found in cord blood and young peripheral blood.^24^ These data reveal that MAIT cells upregulate CD161 and IL‐18R as they exit the thymus or soon after they enter the periphery.

## Factors That Regulate MAIT Cell Development

The identification of a three‐stage development pathway for MAIT cells now allows for a detailed assessment of factors that influence this process. In addition to PLZF, we also showed that microRNAs are critical for MAIT cell development, as mice deficient for a member of the RNase III superfamily, Drosha, have drastically reduced MAIT cells and accumulate stage 1 thymic MAIT cells.^24^ NKT cells were also diminished in these mice, supporting previous data that microRNAs are required for NKT cell development.^54^ Future studies should investigate which specific microRNAs are important for MAIT cell development and whether there is overlap with the microRNAs shown to be important for the development of NKT cells (e.g. miR150,^55^ miR‐181 family^56^ and miR17–92 family cluster^57^). Let‐7 microRNAs will be an important candidate to investigate, as they have been shown to directly target the Zbtb16 gene and downregulate PLZF expression in NKT cells.^58^

The loss of IL‐18 and commensal microbiota from GF mice impaired MAIT cell numbers and progression to stage 3 MAIT cells.^24^ A previous study also showed that B cells were not required for MAIT cell development, but were required for peripheral expansion.^16^ Given that thymic precursors for MAIT cells and NKT cells express similar (but not identical) phenotypic markers and both require PLZF and microRNAs for their development, future studies will likely assess whether other factors are common for their development. For instance, several members of the NFkB family of transcription factors have been shown to be important for NKT cell development.^59^ Furthermore, mice deficient for the transcription factors Rorγt, HeLa E‐box binding protein and c‐Myb, have fewer NKT cells as they are unable to rearrange the distal TCR gene segments due to reduced lifespan of DP thymocytes.^59^ Hence, factors that affect thymocyte survival may impact on the development of MAIT cells as the gene encoding the MAIT TCR Vα‐chain (Vα19) is located at the extreme end of the 5′ end of the TCRα locus.^60^ Inhibitors of DNA transcription factors (Id2 and Id3), which interact with E proteins, have also been implicated in the development and differentiation of NKT cells,^61^, ^62^, ^63^, ^64^ and more recently, we showed that members of the linear ubiquitin chain assembly complex, Hoil and Hoip, were required for normal NKT cell development in the thymus.^65^ Interestingly, the SLAM/SAP/Fyn pathway does not appear to be necessary for the development of MAIT cells, whereas it is important for the development of NKT cells.^16^, ^66^, ^67^, ^68^, ^69^ The use of MR1 tetramers in candidate gene knockout mice should lead to the discovery of key molecules that are important for the development of MAIT cells and these can be compared with those that regulate the development of NKT cells.

## MAIT Cell Heterogeneity

At least two functionally distinct subsets of stage 3 MAIT cells exist in mice and these develop following the expression of PLZF.^24^ The major population of MAIT cells in mice expresses RORγt and secretes IL‐17 upon activation, while a smaller subset expresses T‐bet and produces IFN‐γ upon activation.^6^, ^24^ With the exception of PLZF which is high on both these populations, they are reminiscent of mouse PLZF^lo^ T‐bet^hi^ NKT1 cells and PLZF^int^ Rorγt^+^ NKT17 cells, which secrete IFN‐γ and IL‐17, respectively.^70^ Notably, mouse thymus contains a third population of NKT cells, termed NKT2 cells, which are PLZF^hi^ T‐bet^lo^ cells and predominantly produce IL‐4.^70^ While low levels of IL‐4 production were detected upon stimulation of mouse thymic MAIT cells,^6^ a defined population of MAIT cells that are equivalent to NKT2 cells has yet to be identified. In human thymus and peripheral blood, stage 3 MAIT cells coexpress intermediate levels of T‐bet and RORγt and secrete predominantly IFN‐γ and TNF upon activation.^12^, ^18^, ^24^, ^71^, ^72^, ^73^ This differs to stage 3 MAIT cells from naïve mice, which are mutually exclusive for T‐bet and RORγt expression, although intranasal infection of mice with *Salmonella* Typhimurium causes MAIT cells to coexpress these transcription factors. Thus, previously activated MAIT cells in mice appear to more closely resemble their human counterparts.^32^ While very few MAIT cells from human blood appear to produce IL‐17, MAIT cells from other human tissues can secrete IL‐17. For instance, MAIT cells isolated from the female genital tract express more IL‐17 in response to microbial stimuli compared to MAIT cells from peripheral blood.^12^ Moreover, tissue resident MAIT cells isolated from human liver vascular beds were the dominant population of IL‐17 producing T cells from this tissue^14^ and several studies have reported a role for IL‐17 producing MAIT cells in various autoimmune diseases (reviewed in this issue by Rouxel and Lehuen).^74^ Accordingly, mice and humans contain functionally distinct populations of MAIT cells, although the precise molecular mechanisms that underpin the differentiation into each distinct population remain largely unknown.

## Extrathymic Development of MAIT Cells

MAIT cells continue to mature after they exit the thymus. While stage 3 MAIT cells from human and mouse thymus coexpress CD8α and CD8β, many peripheral MAIT cells express CD8α with low or no CD8β.^5^, ^24^, ^52^ These data suggest that CD8αα^+^ MAIT cells are likely derived from CD8αβ^+^ MAIT cells.^18^, ^52^ Moreover, stage 3 MAIT cells from human thymus have a limited capacity to produce cytokines compared to stage 3 MAIT cells from human blood, suggesting they undergo further maturation in the periphery. In support of this, stage 2 MAIT cells could be detected in the cord blood and the peripheral blood from young donors and stage 2 MAIT cells could be detected in the periphery of PLZF null mice, revealing that MAIT cells can exit the thymus as stage 2 cells, prior to further maturation to stage 3 cells in the periphery.^24^ It is currently unclear what factors drive extrathymic development of MAIT cells, whether it is direct exposure to microbial Ags or other environmental signals such as IL‐18 and/or other cytokines.^24^

The variation in MAIT cell frequency between humans and mice highlights important differences in the development and expansion of MAIT cells between these species. Several factors have been proposed to explain these differences. The housing of mice in specific pathogen‐free conditions likely limits their exposure to microbial Ags and, as described above, MAIT cells are drastically reduced in GF conditions.^2^, ^24^ Interestingly, attempts to reconstitute GF mice with monomicrobial flora or human microbiota only recovered MAIT cell numbers to levels akin to mice housed in specific pathogen‐free conditions, thus levels well below those found in humans.^29^, ^45^ In contrast, intranasal inoculation of mice with *Francisella tularensis* or *Salmonella* Typhimurium leads to rapid expansion of MAIT cells within the lungs of infected mice, levels more consistent with those observed in humans.^31^, ^32^ Future studies will likely examine whether mice caught from the wild or obtained from pet shops have elevated MAIT cells due to increased microbial exposure.

## Homeostasis and Expansion of MAIT Cells

Genetic variability may also contribute to differences in MAIT cell frequency. For example, Cui *et al*. showed that Castaneus mice, a strain of mice originally derived from the wild but housed in specific pathogen‐free conditions, had a 20‐fold increase in MAIT cell numbers compared to common laboratory strains.^45^ The higher number of MAIT cells in Castaneus mice was not due to differences in thymocyte survival but rather to an increase in Vα19‐Jα33 transcripts that was linked to a genetic factor mapped to the Castaneus TCRα locus.^45^ Intriguingly, we recently showed that MAIT cells are over‐represented in NKT cell deficient CD1d^−/−^ mice, suggesting that NKT cells and MAIT cells compete for a similar environmental niche (Figure 1).^24^ This has important implications for the field because NKT cell deficient mice are often used to assess the role of NKT cells in disease models and it is unclear how MAIT cells contribute to these diseases.^75^, ^76^ Furthermore, the frequency of MAIT cells and NKT cells in mice is inversed in humans, such that humans have high numbers of MAIT cells and low numbers of NKT cells, while the reverse is true in mice.^4^ Hence, it will be interesting to determine what factors these innate‐like T cells are competing for, and if other cell types are also involved in this interplay. For example, it was recently shown in mice that innate lymphoid cells compete with T cells for a limited supply of IL‐7.^77^ Furthermore, IL‐7 is also required for the homeostasis and survival of mouse NKT17 cells.^78^ In contrast to these findings in mice, we recently demonstrated a direct correlation in the frequency of MAIT cells and NKT cells in blood from the same human donor.^19^ A similar correlation between MAIT cells and NKT cell frequencies was shown in neonates in a recent study by Ben Youssef and colleagues.^25^ Moreover, they also demonstrated a parallel relationship between Vα7.2^+^ and Vα7.2^−^ subsets of CD161^+^ T cells and while the latter population did not bind MR1 tetramer, their data suggests that both populations undergo a similar postnatal development pathway.^25^

These reports collectively support the concept that MAIT cells, NKT cells and related lineages share environmental maintenance factors, such that when the factors are abundant, these cell populations are increased in blood. Clearly, more work is required to fully appreciate the interactions that maintain innate‐like T cells in the immune system.

**Figure 2:**
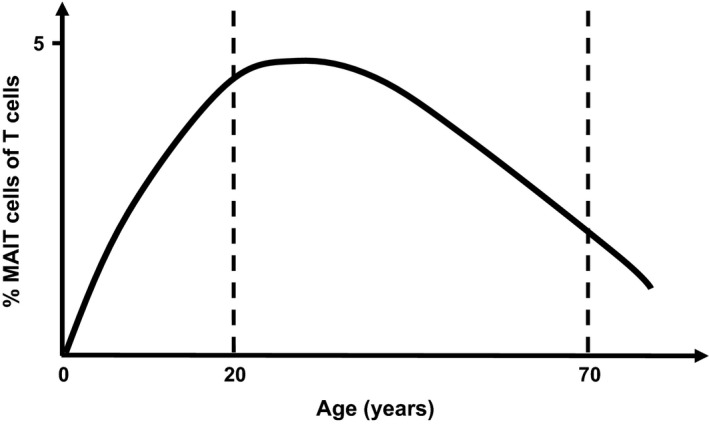
A schematic depicting the percentage of MAIT cells of total T cells through ontogeny.

The high abundance of MAIT cells in humans is likely due to ongoing exposure to microbial stimuli, which drives oligoclonal expansion of MAIT cells that have a reduced TCR repertoire.^7^, ^45^, ^79^ The frequency of MAIT cells increases with age such that very few MAIT cells are observed in the cord blood, presumably due to limited microbial exposure in the womb, while the numbers of MAIT cells increase in the blood from neonates and steadily rise until young adulthood (~30 years of age) before declining over subsequent decades^16^, ^18^, ^24^, ^25^, ^35^, ^52^, ^80^ (Figure 2). It is currently unclear how reduced numbers of MAIT cells impacts on the immune system of older individuals, although it is well described that as humans age they become increasingly immunocompromised.^81^, ^82^ Thus, one key factor that underpins the variability of MAIT cells in humans appears to be age, with optimal MAIT cell numbers coinciding with optimal immunological fitness.

Taken together, these data highlight how MAIT cells continue to develop and expand after they exit the thymus and this process is likely dependent on microbial stimuli.

## Concluding Remarks

MAIT cells, on average, represent the most abundant αβ T‐cell population with a single specificity in the human immune system. Understanding MAIT cell development and homeostasis cells may provide important clues as to why MAIT cell numbers vary between individuals (and species). The recent mapping of a three‐stage MAIT cell development pathway in mice and humans is facilitating further studies to determine the factors that govern this process. There appears to be functional overlap of MAIT cells with other innate‐like T‐cell populations and we must carefully assess the role of these different cell types in the context of microbial immunity. It will also be interesting to address how other related T‐cell subsets develop, including atypical MR1‐restricted Vα7.2^−^ T cells and populations of Vα7.2^−^CD161^+^ T cells, that do not bind MR1 tetramer but share similar characteristics with MAIT cells. Future studies into the development and homeostasis of MAIT cells (and related subsets thereof) may lead to the identification of novel targets to modulate the numbers of these cells which would be valuable in settings where they are depleted, potentially leading to a compromised immune system, such as in cancer and HIV patients.

## Conflict of Interest

The authors declare no conflict of interest.
